# Insights into antiretroviral treatment changes in previously naïve patients: results of a Portuguese cohort

**DOI:** 10.1186/1758-2652-13-S4-P14

**Published:** 2010-11-08

**Authors:** C Silva, D Bento, J Rijo, D Alfaiate, I Aldir, C Araújo, H Farinha, K Mansinho

**Affiliations:** 1 Hospital de Egas Moniz - CHLO, EPE, Infectious Diseases and Tropical Medicine, Lisbon, Portugal; 2 Hospital de Egas Moniz - CHLO, EPE, Pharmacy Department, Lisbon, Portugal

## Purpose of the study

Since the use of more tolerable and less toxic combined antiretroviral (ARV) therapy, most drug-naïve HIV patients achieve viral suppression and immunologic recovery, combined with less AIDS related events. Nevertheless, drug switches are still frequent both as a mean of adherence and toxicity management and as a response to virologic or immunologic failure. The aim of the study was to analyse the number, timing and cause of modifications in the first ARV regimen in order to elucidate more about adverse drug reactions in the current HIV ARV therapy guidelines, indirect signs of adherence and premature virological failure.

## Methods

Non-controlled, observational, retrospective study, based on the clinical files and on a national questionnaire audit for all naïve-patients that began cARV therapy between January 2007 and Mars 2010, followed in an Infectious Diseases Clinic in a central Hospital in Lisbon. SPSS 15.0 was used for statistical analysis.

## Results

During study period, 69 patients of the 285 naïve-patients who started ARV therapy changed their regimen, 64% were male, with a median age of 43 years old. A significant group was born in African Portuguese speaking countries (30%). Most switches occurred on the first 6 months (n=42), 22% on the first month and just 11% after one year of treatment, with more than one modification in 15% of patients. The drug regimen prior to modification included a NNRTI in 62% of the patients. The back-bone regimen included TDF/FTC in 66%, ABC/3TC in 12% and still AZT/3TC in 22% of them. Adverse drug reactions were the most frequent cause of therapy modification (59%), including toxicity in 19 cases and intolerability in 22, reflecting the known side effects of the drugs. Other switch causes were the evidence of virological failure (15%), the simplification of the regimen (10%) and the adjustment during pregnancy (5%). About a fifth of the patients had adherence irregularities. The rate of viral suppression at week 24 of ARV was significantly lower in the group of patients who switched ARV (39% vs 60%; p=0,006), which had also worse immunological recovery at 24 and 48 weeks of ARV.

## Conclusions

Toxicity and intolerability remain the main reasons to change the first ARV regimen, more frequently during the first to sixth months of therapy, witch reinforce the need to evaluate early events that can compromise the adherence, the emergence of resistances and long term toxicity and longevity of this patients.

